# Fine‐Tuning Protein Self‐Organization by Orthogonal Chemo‐Optogenetic Tools

**DOI:** 10.1002/anie.202008691

**Published:** 2021-01-07

**Authors:** Huan Sun, Haiyang Jia, Diego A. Ramirez‐Diaz, Nediljko Budisa, Petra Schwille

**Affiliations:** ^1^ Max Planck Institute of Biochemistry Am Klopferspitz 18 82152 Martinsried Germany; ^2^ Technical University of Berlin Müller-Breslau-Str. 10 10623 Berlin Germany; ^3^ Present address: University of Manitoba 44 DysartRd R3T 2N2 Winnipeg MB Canada

**Keywords:** bottom-up reconstitution, chemo-optogenetic tools, FtsZ, genetic code expansion, membranes, synthetic biology

## Abstract

A universal gain‐of‐function approach for the spatiotemporal control of protein activity is highly desirable when reconstituting biological modules in vitro. Here we used orthogonal translation with a photocaged amino acid to map and elucidate molecular mechanisms in the self‐organization of the prokaryotic filamentous cell‐division protein (FtsZ) that is highly relevant for the assembly of the division ring in bacteria. We masked a tyrosine residue of FtsZ by site‐specific incorporation of a photocaged tyrosine analogue. While the mutant still shows self‐assembly into filaments, dynamic self‐organization into ring patterns can no longer be observed. UV‐mediated uncaging revealed that tyrosine 222 is essential for the regulation of the protein's GTPase activity, self‐organization, and treadmilling dynamics. Thus, the light‐mediated assembly of functional protein modules appears to be a promising minimal‐regulation strategy for building up molecular complexity towards a minimal cell.

## Graphical abstract

We developed orthogonal chemo‐optogenetic tools as a minimal regulation strategy to fine‐tune in‐vitro protein self‐organization with light. Our chemo‐optogenetic tools are designed by orthogonal translation with an expanded genetic code. Such introduced bio‐orthogonal chemical groups operate at the level of single specific amino acids, but perform tight control of enzyme activity and, thereby, the ability of protein self‐organization.

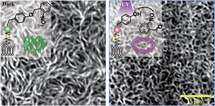

Bottom‐up reconstitution of well‐characterized biological modules in a biomimetic microenvironment allows to explore the mechanisms and molecular origins of key processes of life^[1]^ in a deductive way, and holds tremendous potential for applications from medicine to biotechnology.^[2]^ By minimizing the redundancy and complexity of cellular networks, a large number of biological phenomena have been successfully reconstructed and examined from the bottom up, including pattern formation by reaction‐diffusion,^[3]^ as well as cytoskeletal dynamics of eukaryotic^[4]^ and prokaryotic^[5]^ systems. However, reconstituting the hierarchical order of cellular structures and processes in vitro has been complicated so far by the challenge of controlling both spatial location and temporal activity of the independent biological components with the final aim to link them to specific cellular responses in vivo.

With regard to spatiotemporal control, the use of light to manipulate protein activities is a particularly powerful approach, because the amplitude, wavelength, spatial location, and timing of light illumination can be controlled precisely.^[6]^ Proteins can be spatially targeted and bio‐orthogonally patterned on the membrane by light through genetic fusion, such as light‐inducible chemically modified phospholipid anchors,^[7]^ photoactivatable chemical dimerization,^[8]^ or reversible optogenetic pairs.^[9]^ Moreover, dynamic protein pattern formation can be regulated by photo‐switching the conformation of inhibiting isomeric peptides.^[10]^ These optochemical and optogenetic methods have shown to be sufficient to selectively control pattern formation in vitro. Nevertheless, it would be highly desirable to develop a strategy to directly control spatiotemporal protein activity with even more minimal disruption of protein conformation, which could ideally be biorthogonal and easily transferred to other systems.

Incorporation of noncanonical amino acids (ncAAs) is a particularly promising tool, able to transfer new chemical functions and specific spectroscopic probes into protein structures. With the rapid development of genetic code expansion, numerous photocaged ncAAs^[11]^ have been synthesized and site‐specifically incorporated into proteins of interest via the addition of orthogonal protein biosynthetic machinery.^[12]^ They allow to mask side chain functionalities of site‐specifically incorporated amino acids. These masked substrates can then be non‐invasively unmasked by UV light irradiation revealing critical functionalities. In contrast to approaches based on genetic fusion, the photocaged amino acids containing a single and small light‐cleavable caging group allow for selectively manipulating proteins through removing the caging group under UV light, causing minimal change in protein conformation.^[13]^ They are successfully genetically encoded and widely applied in optical control of protein function and cellular processes.^[14]^ So far, however, none of the reported systems based on photocaged amino acids has been validated to enable rational regulation of reconstituted protein activity and precise control of protein self‐organization on model membranes in vitro.

Herein, we employ site‐specific photocaged non‐canonical amino acids to manipulate cell‐free FtsZ self‐organization on model membranes with light. The reconstituted biological system we focused on is the most well‐known prokaryotic division protein, filamenting temperature‐sensitive mutant Z, abbreviated FtsZ, which is widely conserved in bacteria and a homologue of eukaryotic tubulin. Systematic reconstitution of FtsZ on membranes in vitro from the bottom up is considered to be a promising step towards the assembly of a minimal division machinery. Through masking a key residue of FtsZ via a photocaged tyrosine analog (*ortho*‐nitrobenzyl‐l‐tyrosine, ONBY), we can precisely regulate its GTPase activity, and thus, the GTP hydrolysis‐driven self‐organization and the treadmilling dynamics by protein uncaging with UV light. Without doubt, this represents a potent minimal regulation strategy for the next level of optogenetically controlled spatiotemporal bottom‐up reconstitution towards a synthetic cell. (Figure 1).

**Figure 1:**
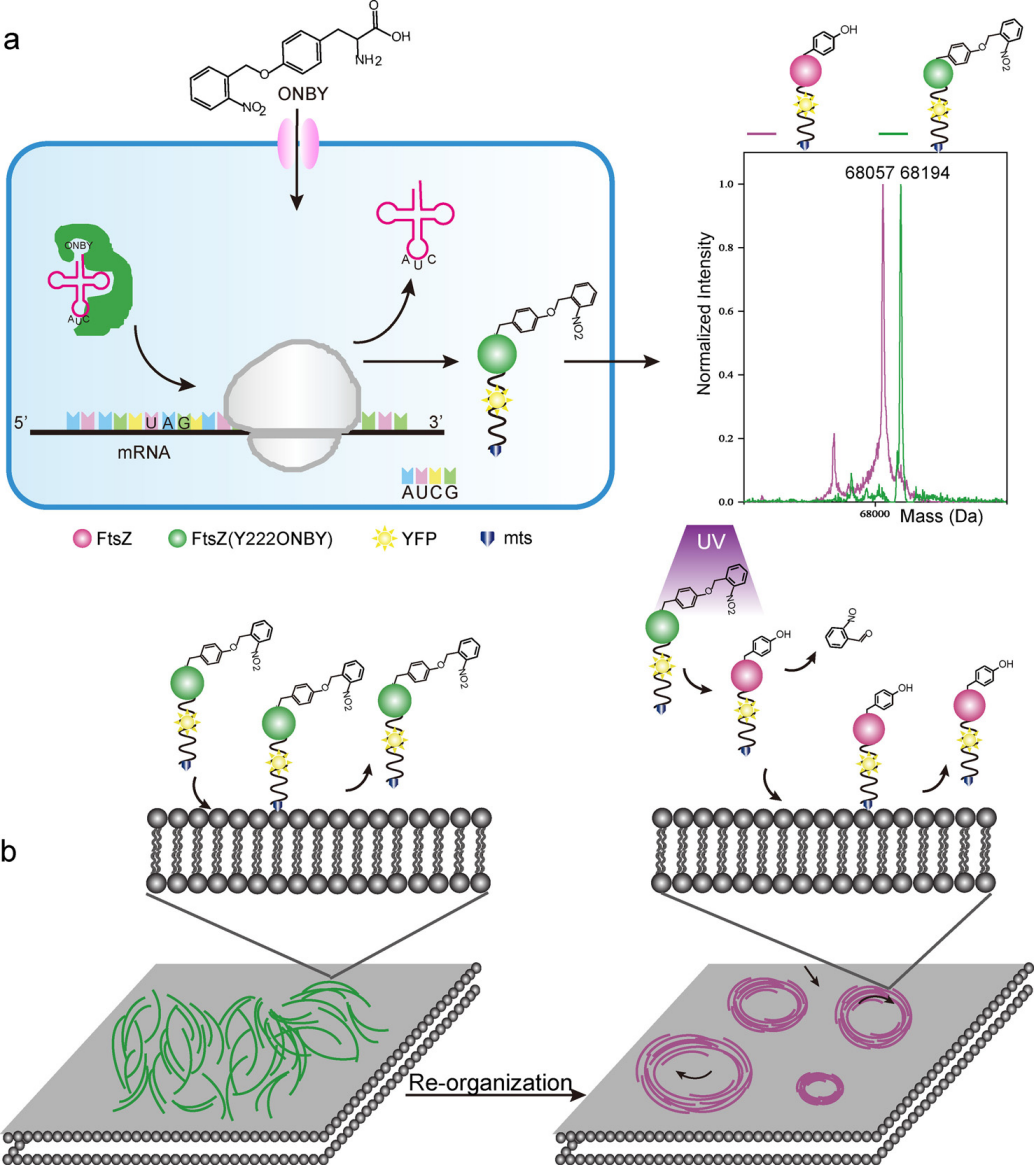
Schematic illustration of site‐specific incorporation of ONBY into FtsZ‐YFP‐mts and photo‐induced protein self‐organization on model membrane. a.) Left: Recombinant expression of site‐specific ONBY incorporated FtsZ‐YFP‐mts at tyro‐sine222 (Y222) position in *Escherichia coli* (*E. coli*). Right: The fidelity of ONBY incorporation into FtsZ was verified by ESI‐MS. Deconvoluted mass: WT‐FtsZ‐YFP‐mts: expected: 68 061.29 Da, observed: 68 057.84 Da; FtsZ(Y222ONBY)‐YFP‐mts: expected: 68 196.2059 Da, observed: 68 194.24 Da. The small peak at 68 166.68 Da represents the reduction of the nitro group to an amine (−30 Da). b.) Schematic illustration showing how filament‐forming, but hydrolysis‐inactive FtsZ(Y222ONBY)‐YFP‐mts is converted into WT‐FtsZ‐YFP‐mts upon UV (365 nm) irradiation by cleavage of the ONB group. As a consequence, photocaged FtsZ self‐organizes into dynamic ring formation in membrane.

To visualize FtsZ binding and self‐assembly on the membrane in real time, the FtsZ mutant FtsZ‐yellow fluorescent protein (YFP)‐membrane‐targeting sequence (mts) was utilized through replacing the FtsZ central hub with a YFP and an amphipathic helix to provide autonomous membrane attachment.^[15]^ This minimal construct has demonstrated to self‐organize and form dynamic ring patterns on supported lipid bilayers (SLB) when monitored by total internal reflection fluorescence (TIRF) microscopy.^[16]^ Tyrosine 222 (Y222) is located at the interdomain region of FtsZ between N‐terminus and C‐terminus (Figure 2 a). It is essential for FtsZ assembly into ring like patterns,^[17]^ representing a suitable key residue to be selected for abolishing/reactivating dynamic FtsZ function. In order to generate light activated FtsZ in the bottom‐up reconstitution system, we targeted Y222 by genetically encoding the incorporation of the photocaged tyrosine analog, ONBY using a highly specific and efficient *Methanococcus jannaschii* tyrosyl‐tRNA synthetase, ONBYRS. This synthetase contains 10 mutations: Y32A, L65A, H70N, G105Q, Q109A, D158S, I159S, L162A, A167S and A180Q.^[18]^ Meanwhile, another tyrosine 339 (Y339) on FtsZ located far to the GTPase activity center was also substituted by ONBY to determine which position is proper to be photocaged. Then the purity and fidelity of ONBY incorporated FtsZ‐YFP‐mts were validated by SDS‐PAGE and ESI‐MS (Figure S1).

**Figure 2:**
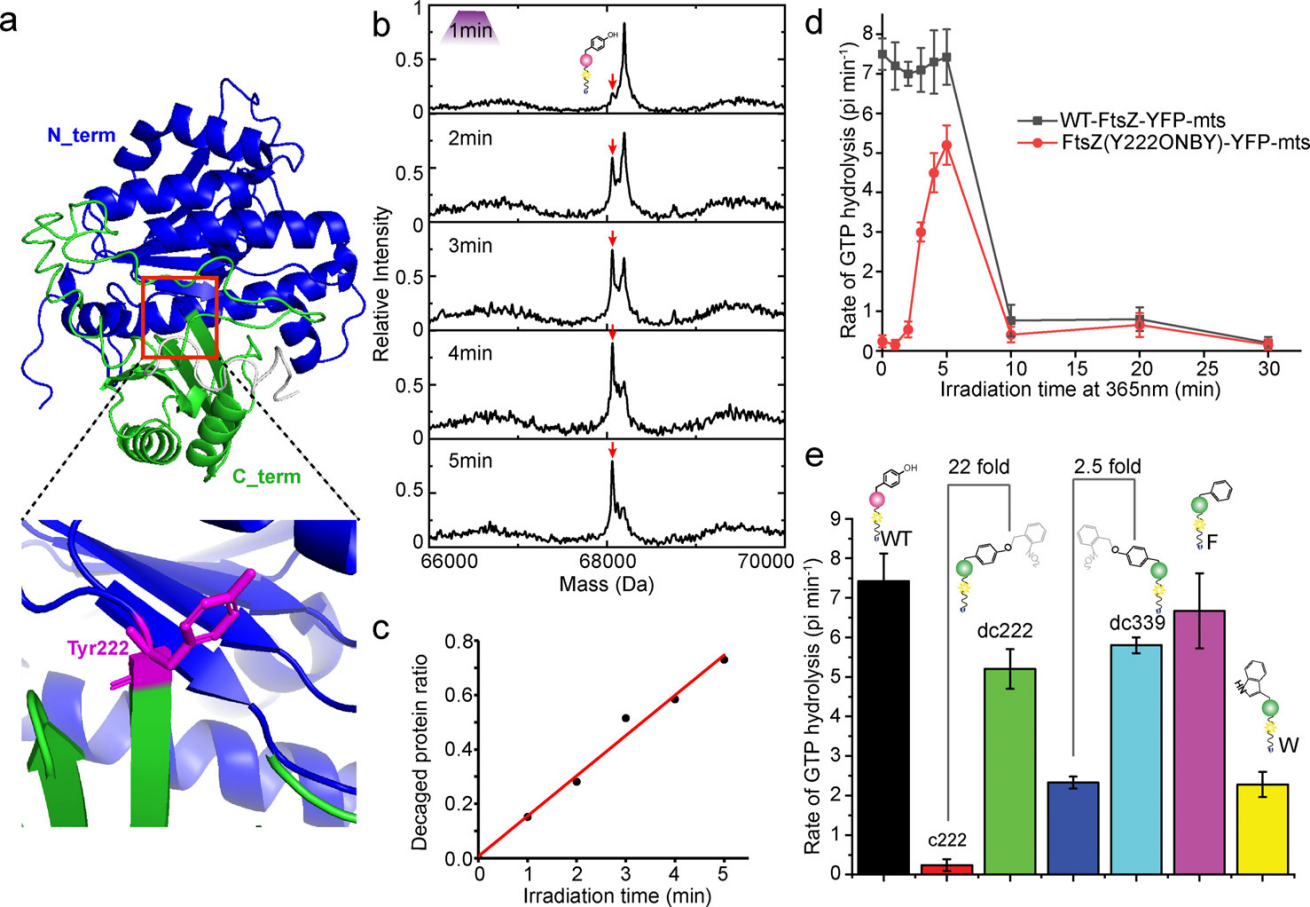
Photo‐activating GTPase activity. a.) Protein structure of FtsZ and the selected position for incorporating the caged tyrosine. b.) ESI‐MS analysis of photolysis of FtsZ(Y222ONBY)‐YFP‐mts with 365 nm for different activation time. Expected ESI‐MS size: WT‐FtsZ‐YFP‐mts: 68 061.29 Da, FtsZ(Y222ONBY)‐YFP‐mts: 68 196.20 Da. Red arrows indicate the uncaged FtsZ. c.) Quantification of uncaged FtsZ (Y222ONBY)‐YFP‐mts protein ratios upon irradiation for different time. The ratio is calculated by the uncaged protein against the total protein. d.) GTPase activity of WT‐FtsZ‐YFP‐mts and FtsZ(Y222ONBY)‐YFP‐mts irradiated with UV at 365 nm for different time. e.) GTPase activity of WT‐FtsZ‐YFP‐mts, FtsZ(Y222/339ONBY)‐YFP‐mts, FtsZ(Y222F)‐YFP‐mts, and FtsZ(Y222W)‐YFP‐mts.

To demonstrate the photolysis of *ortho*‐nitrobenzyl group (ONB), photocaged FtsZ(Y222ONBY)‐YFP‐mts was irradiated with UV light at 365 nm. Upon irradiation for 2 min, there is a new peak representing uncaged FtsZ product observed via MS analysis (Figure 2 b). The photo‐induced cleavage reaction is determined by the light intensity and activation time. To maximize the cleavage efficiency, the light intensity was kept at maximum (15 mW) in our experiment. We find the new peak gradually increase with longer UV illumination time, while the ONB caged FtsZ peak sequentially decreases (Figure 2 b,c). By irradiating for 5 minutes, a conversion efficiency of up to approximately 73 % can be achieved (Figure 2 c). Due to the relatively slow photocleavage process of the ONB group,^[19]^ the ONB blocked FtsZ‐YFP‐mts can't be totally converted to the wild type (WT‐FtsZ‐YFP‐mts). Long time activation may potentially increase the uncaging yield; however, illumination times exceeding 5 min proved to be harmful to protein. Nevertheless, the cleavage efficiency in a short time (5 min) is already sufficient to spatiotemporally abolish blockage and re‐activate FtsZ function. Similarly, the caging group ONB on Y339 can be continuously removed upon UV illumination, while the maximum efficiency that can be achieved is only about 38 % (Figure S2). When checking the ESI‐MS of FtsZ(Y339ONBY)‐YFP‐mts, we found roughly 48 % of protein with ONBY groups were reduced to the non‐photosensitive aminobenzyl mutant that can't be decaged with UV light (Figure S2). As reported, the photo‐cage (nitrobenzyl) groups on amino acids, such as ONBY, are prone to be reduced to amine in *E. coli*,^[20]^ which is influenced by the steric accessibility of the ONBY residue and different sequence contexts.^[14c]^ Additionally, the surrounding microenvironment of ONBY, like the geometry and energy functions,[^14b^, ^21^] could also reduce the cleavage efficiency.

We further investigated the GTPase activity of FtsZ blocked by the photocaged ONB group in response to UV (365 nm) exposure. Careful inspection of Figure 2 d indicates that the incorporation of ONBY at both, Y222 and Y339, efficiently abolished GTPase activity of FtsZ for more than 95 % compared to WT protein (Figure 2 e), which could be due to the large size of the ONB group or the hydroxyl group change. To explore the possible reasons, Y222 was substituted by phenylalanine (FtsZ(Y222F)‐YFP‐mts) and tryptophan (FtsZ(Y222W)‐YFP‐mts). FtsZ(Y222F)‐YFP‐mts demonstrates a similar GTPase activity compared to WT, indicating that removing a hydroxyl group will not reduce the GTP hydrolysis capability. Conversely, GTPase activity of FtsZ(Y222W)‐YFP‐mts is inhibited for 70 % by an indole group, being a bulkier side chain compared to a phenyl group. The results demonstrate that the volume of the amino acid side chain and the bulk at position 222 are important parameters for inhibiting protein activity.

Figure 2 e provides solid proof that the blocked GTPase activity can be promptly rescued by photo‐activation (Figure 2 d), resulting in more than 22‐fold activation. This indicates an excellent OFF to ON switching behavior. The GTPase activity of FtsZ gradually increases with longer exposure time and reaches a maximum after 5 min irradiation. Unfortunately, the enzyme activity dramatically decreases when the protein is irradiated for more than 5 min (Figure 2 d), which is consistent with ESI‐MS analysis of damaged FtsZ, as mentioned before. Additionally, due to the lower cleavage efficiency, GTPase activity of FtsZ(Y339ONBY)‐YFP‐mts can only be enhanced by about 2.5‐fold upon UV treatment (Figure 2 e). Similarly, GTPase activity of FtsZ(Y339ONBY)‐YFP‐mts dramatically decreases for illumination times longer than 5 min (Figure S3).

After demonstrating that we could successfully photo‐induce FtsZ′s GTPase activity, we sought to investigate the self‐organization and dynamic pattern formation of FtsZ on a supported lipid model membrane. FtsZ self‐assembly on SLB was monitored by TIRF microscopy. WT‐FtsZ‐YFP‐mts quickly polymerizes into dynamic bundle structures on the SLB and after several minutes self‐organizes into highly dynamic, small and dim closed circular structures (Figure 3 a, Movie S1). In contrast, as can be seen in Figure 3 b, FtsZ(Y222ONBY)‐YFP‐mts without UV treatment forms a chaotic mesh of thick filament bundles that entirely covers the membrane area (Movie S2). There is no distinctive dynamic ring formation, illustrating the successful abolishment of energy dissipating FtsZ self‐organization. Interestingly, in spite of a high‐level blockage of enzymatic activity, FtsZ(Y339ONBY)‐YFP‐mts and FtsZ(Y222W)‐YFP‐mts can still form ring patterns with similar morphology as WT, indicating that blocking the ability of GTP catalysis alone is not sufficient for structural control of FtsZ ring formation in vitro (Figure 3 b).

**Figure 3:**
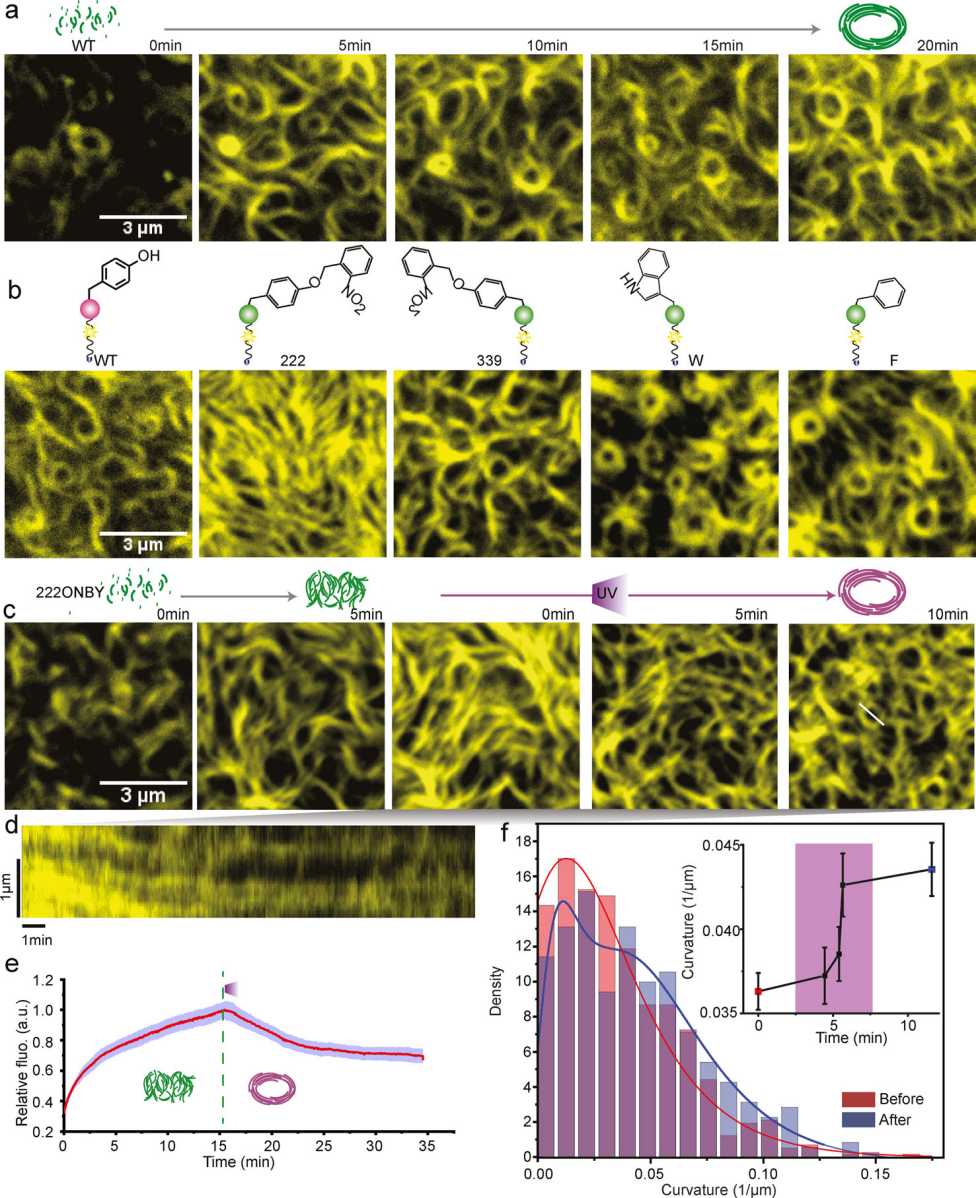
Photo‐control of FtsZ self‐organization on supported lipid bilayer. a.) Snapshots showing dynamic cytoskeletal patterns of WT‐FtsZ‐YFP‐mts emerging on a supported membrane (0.5 μM WT‐FtsZ‐YFP‐mts, 4 mM GTP and 1 mM Mg^2+^). Scale bar, 3 μm. b.) Representative images of cytoskeletal patterns of WT‐FtsZ‐YFP‐mts, FtsZ(Y222/339ONBY)‐YFP‐mts, FtsZ(Y222F)‐YFP‐mts and FtsZ(Y222W)‐YFP‐mts. c.) Dynamically controlling ring pattern formation of FtsZ(Y222ONBY)‐YFP‐mts on SLB by uncaging ONB group with 365 nm UV light (0.5 μM WT‐FtsZ(Y222ONBY)‐YFP‐mts, 4 mM GTP and 1 mM Mg^2+^). Scale bar, 3 μm. d.) The kymograph illustrates the representative ring formation during photo‐activation. The white line in c. indicates the position for the kymograph analysis. e.) The averaged fluorescence intensity of FtsZ on membrane illustrates changes of the protein density upon UV activation. f.) The curvature distribution of FtsZ patterns on membrane before and after UV activation (before: N=617; after: N=384). Inset: the change in curvature over time (mean±SE, N>270). The solid curves represent the extreme fitting.

Since FtsZ(Y222ONBY)‐YFP‐mts demonstrates unique inhibition features for both enzyme activity and ring pattern formation, we investigate this mutant more closely, by employing its dynamic OFF‐to‐ON switching property to globally light‐trigger FtsZ ring formation on membrane in vitro. As expected, under light exposure, light‐induced uncaging of the ONB group can enhance GTPase activity and rescue the dynamics of FtsZ self‐organization. In this process, the dense filament bundles of FtsZ(Y222ONBY)‐YFP‐mts gradually become more curved, and over time organize into dynamic chiral vortices (Figure 3 c,d, Movie S3). Intriguingly, we find that increasing GTPase activity upon light activation reduces the protein density on the membrane by about 30 % (Figure 3 e). When the catalysis rate reaches a level that is comparable with WT, the overall protein structures also resemble WT morphology and dynamics. This indicates that protein dynamics play an important role in controlling protein density on the membrane, further regulating divisiome formation. Interestingly, the curvatures of FtsZ patterns change over time upon light activation (Figure 3 f), indicating that there is indeed a structural rearrangement within the filaments during GTP hydrolysis as stated by structural studies.^[22]^ Therefore, through the tight control of protein activity by light induction, we can regulate protein density on the membrane and further control protein self‐organization.

In the next step, we further investigated the morphology of FtsZ rings. No big difference is found between the photo‐reactivated FtsZ and WT when determining the average diameters of formed rings of about 0.8±0.1 μm (Figure 4 a). This is similar to the Z ring diameter in *E. coli* cells (0.7–1.4 μm).^[23]^ FtsZ(Y222F)‐YFP‐mts also maintains similar ring size with WT, while FtsZ(Y222W)‐YFP‐mts shows a significant smaller size (0.6±0.1 μm), that is, an about 40 % reduction when compared to WT. Besides the size, we also investigated the ring rotation velocity by calculating the slopes of kymographs (Figure 4 c) generated along the circumference. As displayed in Figure 4 c, the velocity distributions for WT‐FtsZ‐YFP‐mts and uncaged FtsZ(Y222ONBY)‐YFP‐mts are comparable. The mean velocity of WT is 20.3±5.9 nm s^−1^, while photo‐activated FtsZ exhibits a slightly lower rotational speed (17.1±4.7 nm s^−1^), which is reasonable because of the non‐complete uncaging of the ONB group in FtsZ(Y222ONBY)‐YFP‐mts upon UV treatment. Low GTPase activity can slow down the treadmilling dynamics of FtsZ self‐organization^[24]^ leading to slightly reduced rotational velocity. As mentioned before, the phenylalanine mutant shows no significant reduction in GTPase activity, while the tryptophan mutant can significantly decrease GTP hydrolysis of FtsZ. As a result, FtsZ(Y222F)‐YFP‐mts with similar GTPase activity (6.7±0.9 pi min^−1^) as WT rapidly self‐organized into homogenous and classical vortices (velocity: 20.2±5.7 nm s^−1^), while the GTPase‐deficient FtsZ(Y222W)‐YFP‐mts (2.3±0.3 pi min^−1^) polymerized into rings of much reduced dynamics (velocity: 14.2±4.1 nm s^−1^) (Figure 4 c). These velocity analyses further verify that the aromatic benzene ring and proper residue's side chain space regulate FtsZ′s ability of self‐organization into dynamic treadmilling vortices. In contrast to FtsZ(Y222ONBY)‐YFP‐mts, FtsZ(Y339ONBY)‐YFP‐mts can still form similar ring patterns compared to WT, but with lower velocity (13.6±2.0 nm s^−1^) due to the reduced GTPase activity (Figure 4 d). Interestingly, upon UV activation the ring dynamics was accelerated and the velocity was increased to 17.4±5.8 nm s^−1^ (Figure 4 d), confirming that GTP hydrolysis is directly linked to treadmilling.[^16^, ^25^]

**Figure 4:**
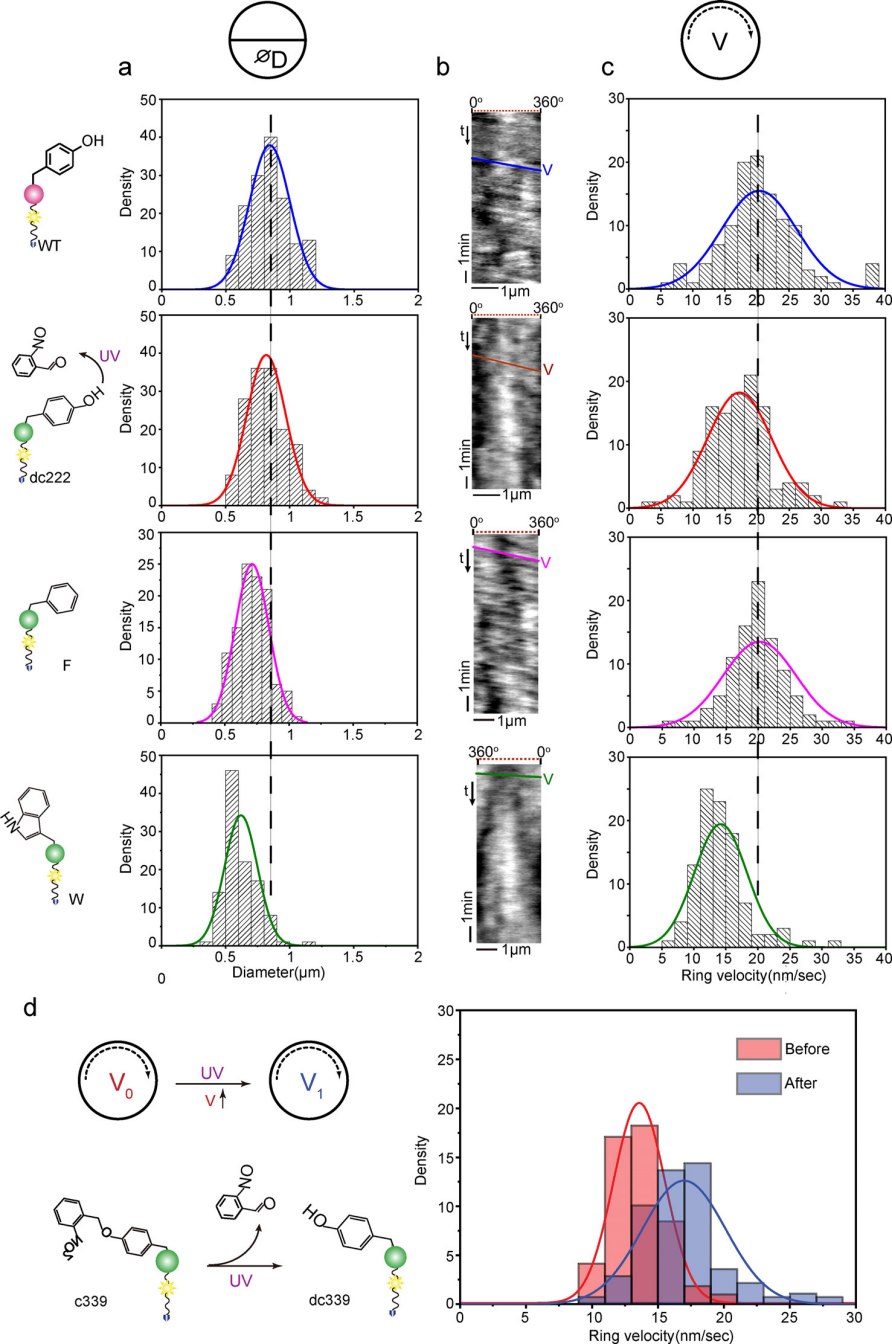
Photo‐activated FtsZ(Y222/339ONBY)‐YFP‐mts self‐organizes into vortices with proper size and treadmilling speed. a.) Ring size distributions of WT‐FtsZ‐YFP‐mts (N=150), uncaged FtsZ(Y222ONBY)‐YFP‐mts (N=150), FtsZ(Y222F)‐YFP‐mts (N=100) and FtsZ(Y222W)‐YFP‐mts(N=100). Diameters were determined by measuring the peak‐to‐peak distance with an intensity plot profile.^[11]^ b.) Representative kymographs along the circumference of vortices. The respective slopes correspond to the treadmilling velocity of the vortices. c.) Velocity distributions of ring patterns (N=100). d.) Scheme and velocity distributions of ring patterns formed by FtsZ(Y339ONBY)‐YFP‐mts before (*n*=174) and after (*n*=139) UV activation. The solid curves in a. c. and d. represent the Gauss fitting.

In summary, we have demonstrated that the functional dynamics of FtsZ, leading to treadmilling rings on membranes upon GTP hydrolysis, can be made light‐switchable through site‐specific incorporation of a photocaged tyrosine analog. Protein activity can be blocked with minimal modification, through introducing a single photocaged group at an essential position, and further be efficiently switched on by light. The dynamic self‐organization of FtsZ on membranes in vitro can herein be tightly controlled through removing the caging group, which allows reverting catalysis, morphology and dynamics of caged proteins without damage. Unlike the traditional methods employed for studying FtsZ (e.g., mutagenesis), the optogenetic strategies developed here allow for acute restoring of protein self‐assembly, enabling a fine‐tuning of activity in situ, dependent on the intensity and duration of illumination. Furthermore, our genetically encoded, light controllable FtsZ can be transferred into bacterial cells to dissect cell division mechanisms. Not limited to the FtsZ system, our minimal regulation strategy can also be extended to other reconstituted systems, opening up great perspectives for the development of well‐controlled minimal systems towards a synthetic cell.

In the future, we envision a further development of even more efficient photo‐caged amino acids using a combination of creative chemistry and directed evolution of enzymes suitable for use in both prokaryotic and eukaryotic cells, as well as in sophisticated in vitro reconstitution assays. Ideally, it will be possible to control protein functions with the precision of UV light radiation over a wide range of spatial and temporal scales. In this way, orthogonal translation will expand the growing set of chemo‐optogenetic tools.^[26]^

## Conflict of interest

The authors declare no conflict of interest.

## Supporting information

As a service to our authors and readers, this journal provides supporting information supplied by the authors. Such materials are peer reviewed and may be re‐organized for online delivery, but are not copy‐edited or typeset. Technical support issues arising from supporting information (other than missing files) should be addressed to the authors.

Supplementary

Supplementary

Supplementary

Supplementary
