# Supplementary material for: Fine‐Tuning Protein Self‐Organization by Orthogonal Chemo‐Optogenetic Tools
Source: Angew Chem Int Ed Engl. 2021 Jan 7;60(9):4501–6. doi: 10.1002/anie.202008691 (PMC7986231; doi:10.1002/anie.202008691)
Supplement: Supplementary file 1 — Supplementary [file ANIE-60-4501-s003.pdf]

Angewandte Chemie
Eine Zeitschrift der Gesellschaft Deutscher Chemiker

Supporting Information

Fine-Tuning Protein Self-Organization by Orthogonal Chemo-Optogenetic Tools

Huan Sun[+], Haiyang Jia[+], Diego A. Ramirez-Diaz, Nediljko Budisa, and Petra Schwille**

anie_202008691_sm_miscellaneous_information.pdf
anie_202008691_sm_Movie_S1.avi
anie_202008691_sm_Movie_S2.avi
anie_202008691_sm_Movie_S3.avi

Supporting Information

Supplementary Methods

Protein expression and purification

WT-FtsZ-YFP-mts, FtsZ(Y222F)-YFP-mts, and FtsZ(Y222W)-YFP-mts were cloned in pET-11b expression vector and transformed into *Escherichia coli* (*E. coli*) strain BL21(DE3). Proteins were induced by 0.5mM IPTG at 20°C for overnight. The genes of FtsZ(Y222ONBY)-YFP-mts and FtsZ(Y339ONBY)-YFP-mts containing an amber codon at Y222 and Y339 position were cloned on pET-28a expression vector and co-transformed with ONBYRS cloned on pULTRA into RF1 free BL21(DE3) (B95.ΔA)[1]. A preculture (1-50mL LB with the appropriate antibiotics and 1% glucose) was grown overnight. 1:50 or 1:100 of preculture and was transferred to TB medium. Afterwards, culture was shaken at 37 °C until OD_{600} reaching around 1. Then 1mM *ortho*-nitrobenzyl-L-tyrosine (ONBY, Activate Scientific, Prien, Germany) was added directly into the culture before the target gene induction. Then the culture was shaken continuously for another 30-40min to allow dissolving and cellular uptaking of the ONBY. Protein expression was induced by 0.3mM IPTG for 3.5h at 37 °C. Then FtsZ chimeric proteins were purified as previously described[2]. Briefly, protein was precipitated from the supernatant by adding 30% ammonium sulphate at 4˚C. Afterwards, the precipitate was shaken for 20min at 4˚C at slow speed. After centrifugation and resuspension of the pellet, the protein was purified by anion exchange chromatography using a 5×5 ml Hi-Trap Q-Sepharose column (GE Healthcare, 17515601). The fidelity and purity of the protein was confirmed by SDS-PAGE and mass spectrometry.

Mass-spectrometry

The molecular weight of purified proteins was analyzed on LC-ESI-MS using Agilent 6530 QTOF instrument (Agilent, Santa Clara, CA, USA) after external calibration coupled with an Agilent 1260 HPLC system. 80-100μL of a protein solution with a concentration around 0.1mg/mL was injected for one run. First, proteins were pre-separated on a C5 column (Supelco Analytical, Sigma-Aldrich, Taufkirchen) by reversed-phase chromatography with a gradient elution starting with 5% acetonitrile w/0.1% formic acid in the water, and going to 80% acetonitrile w/0.1% formic acid in water. Detection was carried out with a UV-Vis detector by measurement of A_{280}. Later, the separated protein was transmitted to the mass spectrometer (Thermo Scientific, Waltham, MA, USA). The protein was ionized via electrospray ionization (ESI). Ion analysis was performed with an orbitrap analyzer. The obtained spectrum was deconvoluted with the program MagTran to obtain the molecular mass.

GTPase activity assay of FtsZ-YFP-mts

GTPase activities of FtsZ-YFP-mts was determined by measuring released inorganic phosphate using BIOMOL® GREEN assay (Enzo Life Sciences USA). GTP hydrolysis reaction was performed in polymerization buffer (50mM Tris/HCl, 300mM KCl, 5mM Mg^{2+}, pH 7.5) using FtsZ-YFP-mts at 5μM with 1mM GTP. Reactions were performed every 20sec for a total time of 120sec. After 30 minutes of incubation with BIOMOL® GREEN at room temperature, the absorbance of the samples at 620nm was monitored by TECAN plate reader. The phosphate release concentrations were calculated based on a phosphate standard curve.

Small unilamellar vesicles (SUVs) preparation

1,2-dioleoyl-sn-glycero-3-phosphocholine (DOPC):1,2-dioleoyl-sn-glycero-3-phospho-(1'-rac-glycerol) (DOPG), 70:30mol % mixture, was dissolved in

chloroform in a quartz container, then dehumidified under a gas nitrogen stream. Chloroform traces were further removed through desiccation (1h). Afterward, the lipid film was hydrated to a final concentration of 4mg/ml in supported lipid bilayers (SLB) buffer (50mM Tris-HCl at pH 7.5, 150mM KCl), and incubated at 37°C for 30min. The lipid film was then completely resuspended by vortexing to obtain multilamellar vesicles of different sizes. This mixture was then placed in a bath sonicator where shear forces help to reduce the size of the vesicles, giving rise to small unilamellar vesicles (SUVs). The SUV dispersion was stored at -20°C as 20µl aliquots.

SLBs preparation

Glass coverslips (1.5#, 24x24mm) were cleaned by piranha solution overnight, followed by extensive washing with Milli-Q H_2O. Then glass coverslips were blown dry with compressed air. The reaction chamber was prepared by attaching a plastic ring on a cleaned glass coverslip using ultraviolet glue (Thorlabs No. 68). For supported lipid bilayer formation, the SUV dispersion was diluted in SLB buffer to 0.5$mgml^{-1}$, of which 75µl was added to the reaction chamber. Adding $CaCl_2$ to a final concentration of 3mM induced fusion of the vesicles and the formation of a lipid bilayer on cover slide. After 20min of incubation at 37°C, the sample was rinsed with 2mL pre-warmed SLB buffer.

Self-organization assays

FtsZ-YFP-mts was added to the reaction buffer above the supported lipid membrane in the chamber. The final volume of a sample was approximately 250µl. FtsZ-YFP-mts was added with a final concentration of 0.5µM. Polymerization was induced by adding 4mM GTP.

Total internal reflection fluorescence microscope (TIRFM) imaging

All experiments were performed on a WF1 GE DeltaVision Elite total internal reflection fluorescence microscope (TIRFM, GE Healthcare Life Sciences,

Germany) equipped with an OLYMPUS 100×TIRF objective (NA 1.49). The UltimateFocus feature of DeltaVision Elite maintains the focus plane constant in time. FtsZ-YFP-mts was excited with a 488nm diode laser (10mW, before objective). Fluorescence imaging was performed using a standard FITC filter set. Images were acquired with a PCO sCMOS 5.5 camera (PCO, Germany) controlled by the softWoRx Software (GE Healthcare Life Sciences, Germany). For time-lapse experiments, images were acquired every 3s with a 0.05 s exposure time and light illumination shuttered between acquisitions.

Ring velocity analysis and processing

FtsZ ring velocities were analyzed according to the published approach[3]. Briefly, image analysis was carried out in MATLAB 2016s (MATLAB and Image Processing and Computer Vision Toolbox Release 2016a, The MathWorks, Inc., Natick, Massachusetts, USA) and processing with Fiji/ImageJ. Images corresponded to an average of 5–10 frames from a time-series experiment. For the kymograph analysis, time-series acquisitions were filtered using a standard mean filter (2 pixels) and were drift corrected (multistackreg plugin). A MATLAB script allowed the user to define a ring by providing two coordinates. Every ring was automatically fitted to a circle with radius r. Then, three trajectories corresponding to three concentric circles having radii r, r+1, and r−1 pixels were determined. At this point, the script read the time-series data and calculated a kymograph for each time point and trajectory. To automatically calculate the slope, the kymograph was smoothed with a Savitzky-Golay filter of order 2 and enhance its contrast using a contrast-limited-adaptive-histogram-equalization (CLAHE) routine (MATLAB). Next, using Fourier analysis from previous study[3], the characteristic frequency for the patterns on the kymograph was found. Finally, the slope corresponded to the change in phase at this frequency. Quality criteria were properly chosen to reject low-quality regions over the kymograph. To synchronize time-lapse acquisitions, the initial frame (time 0) was defined when surface mean intensity was around 200 A.U.

Curvature analysis

The curvatures of FtsZ network were analyzed with Stretching Open Active Contours (SOAX)[4]. The following parameters were used. Rigid threshold: 0.01; foreground:0-65535; snake point spacing:1pixel; Minimal snake length:10 pixels; Maximum iterations 10000; change threshold: 0.1 pixels; other parameters: default setting of SOAX.

Supplementary Results

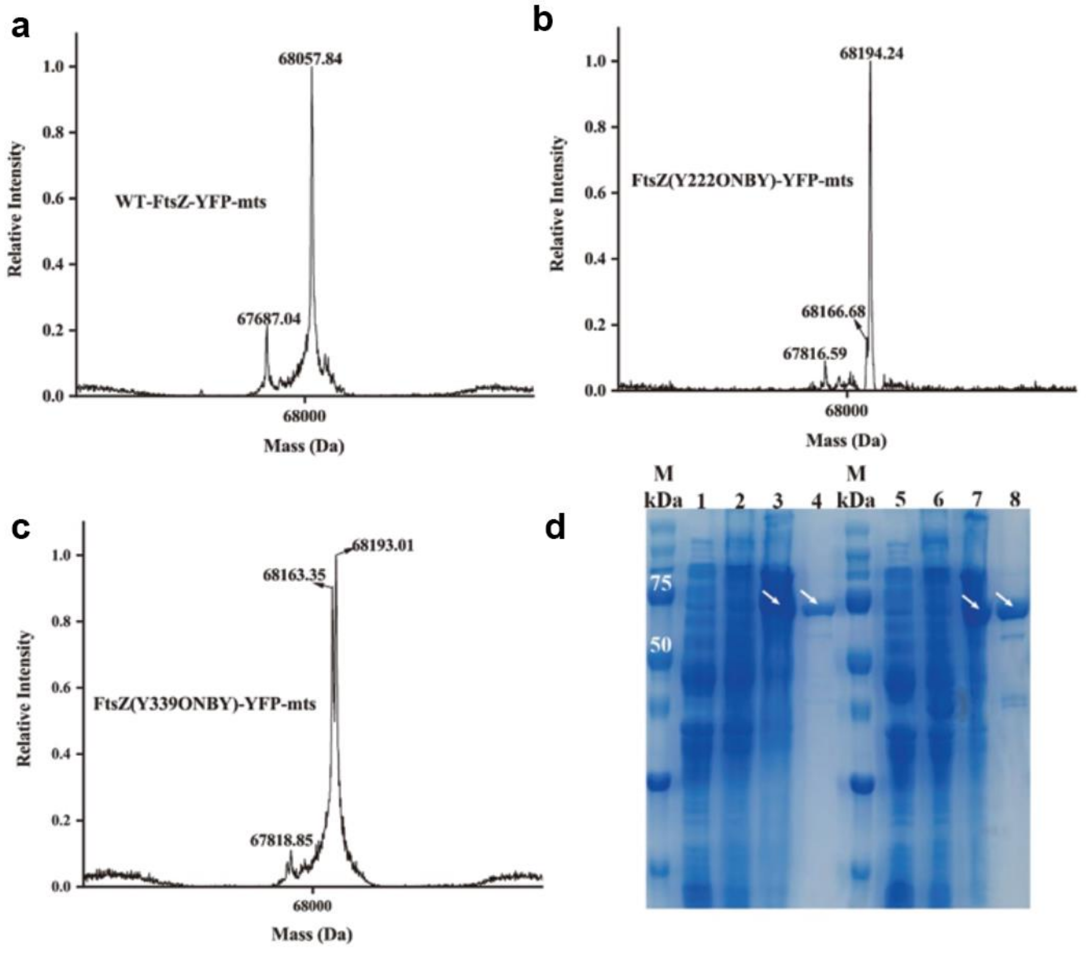

Figure S1 Genetic incorporation of photocaged tyrosine (ONBY) into FtsZ-YFP-mts in *E. coli*. Deconvoluted ESI-MS: (a) WT-FtsZ-YFP-mts: expected: 68061.29Da, observed: 68057.84Da (b) FtsZ(Y222ONBY)-YFP-mts: expected: 68196.2059Da, observed: 68194.24Da. The small peak: 68166.68Da

represents the reduction of the nitro group to an amine (-30Da) (c) FtsZ(Y339ONBY)-YFP-mts: expected: 68196.2059Da, observed: 68193.01Da. The peak: 68163.35Da represents the reduction of the nitro group to an amine (-30Da). The small peaks 67687.04Da, 68166.68Da and peak 68163.35Da which was left-shifted (-375Da) compared with the main peaks exist in WT-FtsZ-YFP-mts, FtsZ(Y222ONBY)-YFP-mts, and FtsZ(Y338ONBY)-YFP-mts, respectively. Those peaks were assigned to the C-terminus (mts region) loss from FtsZ-YFP-mts during MS measurements. The mts adapted from MinD is positively charged[5], while the ESI-MS measurement is under acidic condition. The acidic condition may make mts at a low ionic strength condition. Previously reported, at low ionic strength condition, the N-terminal or C-terminal α-helix is prone to loss[6]. (d) SDS-PAGE with Coomassie staining shows that ONBYRS specifically incorporated ONBY into FtsZ-YFP-mts at Y222 or Y339 position in *E. coli* by suppression of the amber codon: lane 1 and lane 5: ni: non-induced sample; lane 2 and lane 6: induced sample without ONBY addition. The cells were transformed with FtsZ-YFP-mts plasmid bearing amber codon at Y222 or Y339 position, respectively; lane 3 and lane 7: induced sample supplied with ONBY. The cells were transformed with FtsZ-YFP-mts plasmid bearing amber codon at Y222 or Y339 position, respectively; lane 4 and lane 8: purified FtsZ-YFP-mts containing ONBY at Y222 or Y339 position. The full length of FtsZ chimera protein is ~68 kDa. Coomassie-stained bands representing the expected full-length FtsZ-YFP-mts variants were framed by a white arrow.

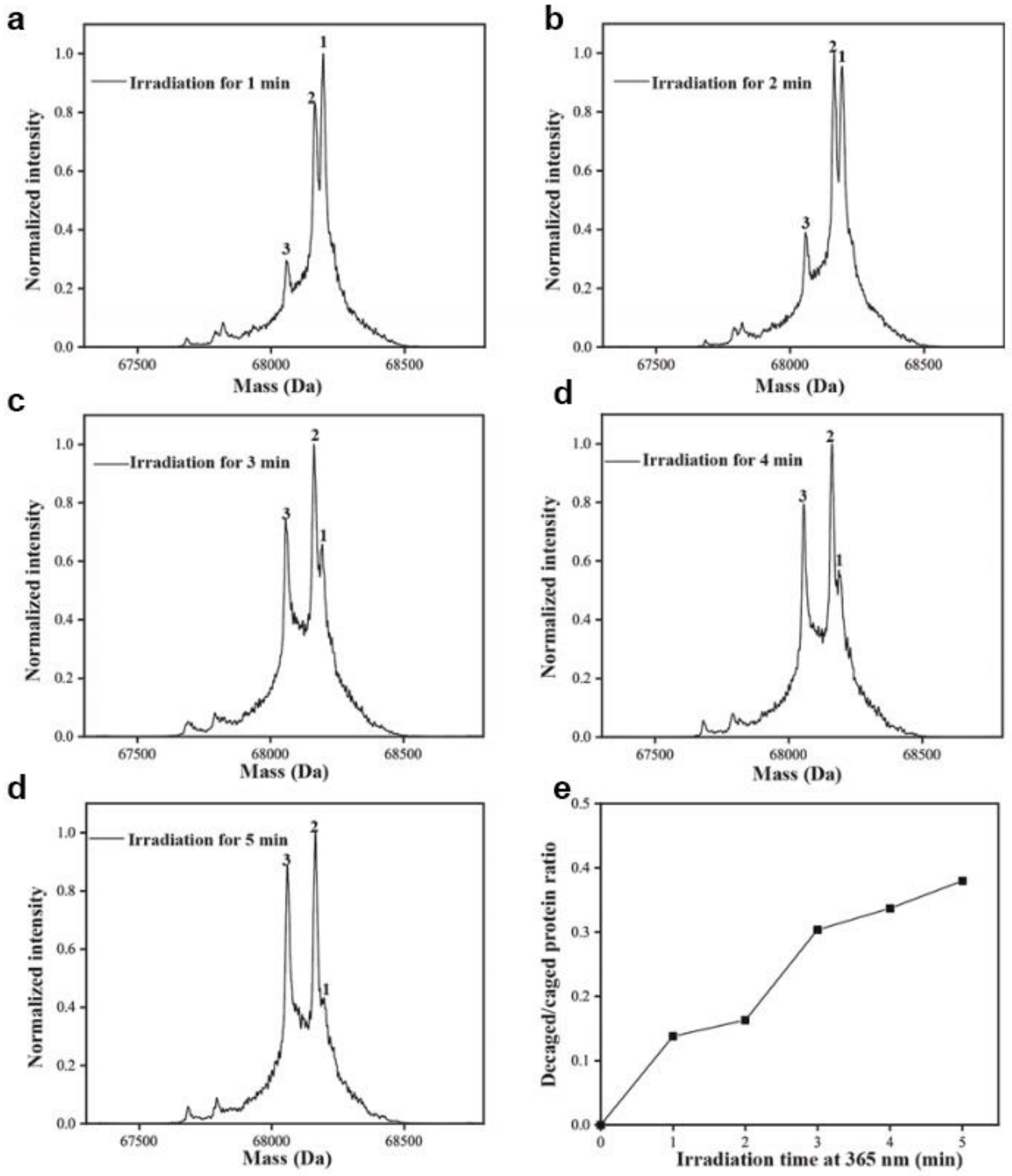

Figure S2 ESI-MS analysis of FtsZ(Y339ONBY)-YFP-mts irradiated at 365nm for different time. Peak 1 in Figure (a-e) represent FtsZ(Y339ONBY)-YFP-mts; peak 2 in Figure (a-e) show FtsZ(Y339ONBY)-YFP-mts with nitro reduced to amine (-30Da); peak 3 in Figure (a-e) represent photo-caged chimera converted to WT-FtsZ-YFP-mts by removing ONB group with UV treatment for 1min (a), 2min (b), 3min (c), 4min (d) and 5min (e). Deconvoluted ESI-MS: FtsZ(Y339ONBY)-YFP-mts: expected: 68196.20Da. Observed: (a) peak 1: 68194.12Da; (b) peak 1: 68194.06Da; (c) peak 1: 68194.30Da; (d) peak 1:

68194.10Da; (e) peak 1: 68194.06Da. FtsZ(Y339ONBY)-YFP-mts with nitro group reduced to amine: expected: 68166.20Da. Observed: (a) peak 2: 68163.53Da; (b) peak 2: 68164.53Da; (c) peak 2: 68163.63Da; (d) peak 2: 68164.59Da; (e) peak 2: 68164.60Da. WT-FtsZ-YFP-mts: expected: 68061.29Da, Observed: (a) peak 3: 68057.03Da; (b) peak 3: 68057.82Da; (c) peak 3: 68058.26Da; (d) peak 3: 68057.03Da; (e) peak 3: 68058.62Da.(f) Quantification of decaged/caged FtsZ (Y339ONBY)-YFP-mts protein ratios upon irradiation for different time. The normalized ESI-MS intensity of WT-FtsZ-YFP-mts, FtsZ(Y339ONBY)-YFP-mts with nitro group reduced to amine and FtsZ(Y339ONBY)-mts were set as x, y and z, respectively. The formula of decaged/caged FtsZ (Y339ONBY)-YFP-mts protein ratio is x/(x+y+z).

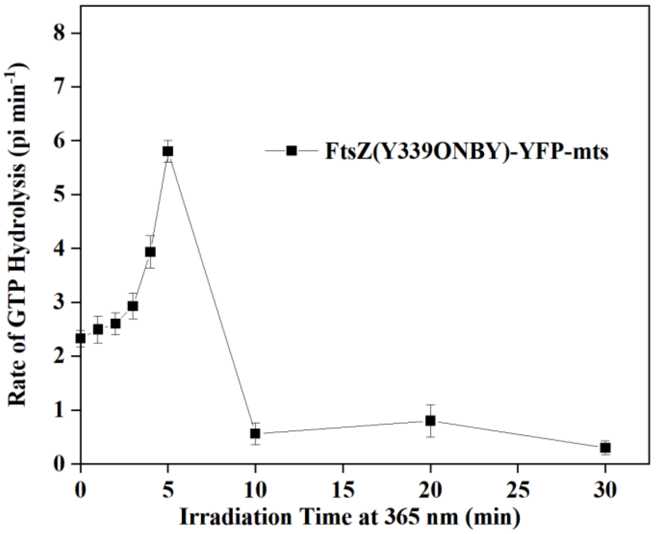

Figure S3 GTPase activity of FtsZ(Y339ONBY)-YFP-mts irradiated with UV at 365nm for different time

Supplementary Movies

Movie S1 Dynamic ring patterns of WT-FtsZ-YFP-mts emerging on a supported membrane (0.5µM WT-FtsZ-YFP-mts, 4mM GTP and 1mM Mg^{2+}).

Movie S2 Dynamic cytoskeletal patterns of FtsZ(Y222ONBY)-YFP-mts (0.5µM FtsZ(Y222ONBY)-YFP-mts, 4mM GTP and 1mM Mg^{2+}).

Movie S3 Switching ring pattern formation of FtsZ(Y222ONBY)-YFP-mts on SLB by decaging ONB group with 365nm UV light (0.5µM WT-FtsZ(Y222ONBY)-YFP-mts, 4mM GTP and 1mM Mg^{2+}).

References

[1] T. Mukai, H. Hoshi, K. Ohtake, M. Takahashi, A. Yamaguchi, A. Hayashi, S. Yokoyama, K. Sakamoto, *Sci. Rep* **2015**, *5*, 1-9.

[2] M. Osawa, H. P. Erickson, *Meth. Enzymol* **2009**, *464*, 3-17.

[3] D. A. Ramirez-Diaz, D. A. García-Soriano, A. Raso, J. Mücksch, M. Feingold, G. Rivas, P. Schwille, *PLoS Biol.* **2018**, *16*, e2004845.

[4] T. Xu, D. Vavylonis, F.-C. Tsai, G. H. Koenderink, W. Nie, E. Yusuf, I.-J. Lee, J.-Q. Wu, X. Huang, *Sci. Rep.* **2015**, *5*, 9081.

[5] L. D. Renner, D. B. Weibel, *J. Biol. Chem.* **2012**, *287*, 38835-38844.

[6] R. M. Caprioli, A. Malorni, G. Sindona, *Mass spectrometry in biomolecular sciences, Vol. 475*, Springer Science & Business Media, **2012**.
